# Supplementary material for: Crawling and Gliding: A Computational Model for Shape-Driven Cell Migration
Source: PLoS Comput Biol. 2015 Oct 21;11(10):e1004280. doi: 10.1371/journal.pcbi.1004280 (PMC4619082; doi:10.1371/journal.pcbi.1004280)
Supplement: S1 Code — (ZIP) [file pcbi.1004280.s012.zip › release/tst/doc/html/globals_defs.html]

Tissue Simulation Toolkit: File Members


Tissue Simulation Toolkit
 0.1.4.1


- Main Page
- Namespaces
- Classes
- Files

- File List
- File Members

- All
- Functions
- Variables
- Typedefs
- Macros

- b
- c
- d
- e
- f
- g
- h
- i
- k
- m
- n
- o
- p
- r
- s
- t
- u
- v
- w
- x
- y
- z

### - b -

- BLACK
  : sticky.h
- BLUE
  : sticky.h
- BOLTZMANN
  : sticky.h

### - c -

- CFILE
  : x11graph.h
- COMMUNICATION
  : sticky.h

### - d -

- DIV
  : sticky.h
- DMUT
  : sticky.h
- DSQR
  : sqr.h

### - e -

- EMPTY
  : sticky.h
- ENDOFSTATES
  : sticky.h
- ENERGYOFFSET
  : sticky.h
- EXTERNAL\_OFF
  : dish.cpp

### - f -

- FAC
  : random.h
- FALSE
  : misc.h
  , output.h
  , sticky.h
- FNAMESIZE
  : output.cpp

### - g -

- GREEN
  : sticky.h
- GRIDX
  : sticky.h
- GRIDY
  : sticky.h

### - h -

- HASHCOLNUM
  : cell.cpp
- HASHCOLPRIME
  : sticky.h
- HOSTDEAD
  : sticky.h

### - i -

- INETSIZE
  : sticky.h
- INIT
  : dish.h
- INITIAL\_BUFSIZE
  : output.cpp
- INP\_PROT
  : sticky.h

### - k -

- KEYBUFSIZE
  : x11graph.cpp

### - m -

- max
  : conrec.cpp
- MAXCELLS
  : sticky.h
- MAXHIST
  : sticky.h
- MAXNEIGH
  : sticky.h
- MAXSEED
  : sticky.h
- MAXTYPE
  : sticky.h
- MBIG
  : random.h
- MEDIUM
  : sticky.h
- MEMORYCHECK
  : warning.h
- MESS\_BUF\_SIZE
  : output.h
- min
  : conrec.cpp
- MOTION
  : x11graph.h
- MSEED
  : random.h
- MZ
  : random.h

### - n -

- NETSIZE
  : sticky.h
- NH\_TH
  : sticky.h
- NHHIST
  : sticky.h
- NOPVM
  : crash.cpp
  , x11graph.cpp
- NULL\_BEAST
  : sticky.h

### - o -

- OK
  : misc.h
  , sticky.h
- OK\_BEAST
  : sticky.h
- OUTFILE
  : x11graph.h

### - p -

- PCO
  : sticky.h
- PLOTPERIODFREQUENCY
  : sticky.h
- PMUT
  : sticky.h
- PMUT2
  : sticky.h
- POTPROT
  : sticky.h

### - r -

- RED
  : sticky.h
- REMARK
  : misc.h
  , sticky.h
- RESIZE
  : x11graph.h

### - s -

- SQR
  : sqr.h
- SWAP
  : x11graph.cpp

### - t -

- TESTCELLS
  : sticky.h
- TIMESTEP
  : qtgraph.h
  , x11graph.h
- TRUE
  : misc.h
  , output.h
  , sticky.h

### - u -

- UNIDENTIFIED
  : warning.h

### - v -

- VERBOSE
  : x11graph.h

### - w -

- WHITE
  : sticky.h

### - x -

- XPM
  : ca.cpp
- xsect
  : conrec.cpp

### - y -

- ysect
  : conrec.cpp

### - z -

- ZYGFILE
  : ca.cpp
- ZYGOTE
  : ca.cpp
- ZYGXPM
  : ca.cpp


---

Generated on Thu Aug 14 2014 22:04:02 for Tissue Simulation Toolkit by  

 1.8.6
